# 2,4-Diphenyl-4,5-dihydro-3*H*-pyrido[2,3-*b*][1,4]diazepine

**DOI:** 10.1107/S1600536809014901

**Published:** 2009-04-30

**Authors:** Hoong-Kun Fun, Chin Sing Yeap, Anita Hazra, Subrata Jana, Shyamaprosad Goswami

**Affiliations:** a X-ray Crystallography Unit, School of Physics, Universiti Sains Malaysia, 11800 USM, Penang, Malaysia; b Department of Chemistry, Bengal Engineering and Science University, Shibpur, Howrah 711 103, India

## Abstract

The asymmetric unit of the title compound, C_20_H_17_N_3_, contains two crystallographically independent mol­ecules (*A* and *B*). In mol­ecule *A*, the two benzene rings form dihedral angles of 74.12 (7) and 7.83 (7)° with the pyridine ring, while in mol­ecule *B* these angles are 77.48 (7) and 21.50 (7)°. The seven-membered heterocyclic ring adopts a boat conformation in both mol­ecules. In the crystal structure, each of the independent mol­ecules forms a centrosymmetric *R*_2_^2^(8) dimer linked by paired N—H⋯N hydrogen bonds. The crystal structure is further stabilized by inter­molecular C—H⋯N hydrogen bonds and C—H⋯π inter­actions.

## Related literature

For bond-length data, see: Allen *et al.* (1987 ▶). For general background and the biological applications of pyridodiazepine compounds, see: Landquist *et al.* (1984 ▶); Smalley *et al.* (1979 ▶); Goswami *et al.* (2009 ▶). For the stability of the temperature controller used for the data collection, see: Cosier & Glazer (1986 ▶).
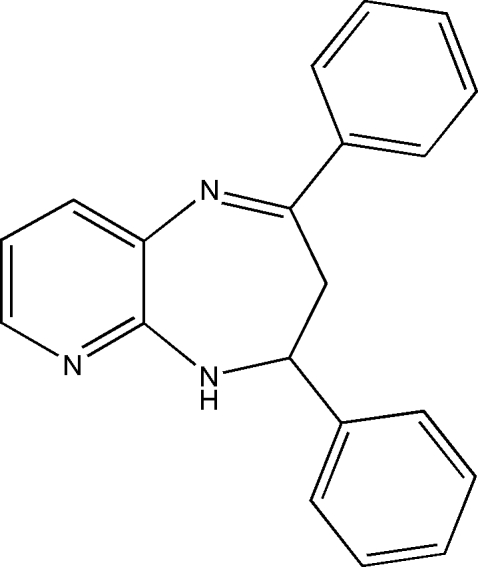


## Experimental

### 

#### Crystal data

C_20_H_17_N_3_
*M* *_r_* = 299.37
Triclinic, 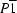

*a* = 5.9969 (3) Å
*b* = 15.3186 (6) Å
*c* = 17.0676 (7) Å
α = 82.588 (3)°
β = 85.266 (2)°
γ = 88.670 (2)°
*V* = 1549.37 (12) Å^3^
*Z* = 4
Mo *K*α radiation
μ = 0.08 mm^−1^
*T* = 100 K
0.50 × 0.33 × 0.05 mm

#### Data collection

Bruker SMART APEXII CCD area-detector diffractometer
Absorption correction: multi-scan (**SADABS**; Bruker, 2005 ▶) *T*_min_ = 0.948, *T*_max_ = 0.996
35167 measured reflections
9044 independent reflections
5978 reflections with *I* > 2σ(*I*)
*R*_int_ = 0.053

#### Refinement

*R*[*F* ^2^ > 2σ(*F* ^2^)] = 0.054
*wR*(*F* ^2^) = 0.133
*S* = 1.08
9044 reflections
423 parameters
H atoms treated by a mixture of independent and constrained refinement
Δρ_max_ = 0.30 e Å^−3^
Δρ_min_ = −0.25 e Å^−3^

### 

Data collection: *APEX2* (Bruker, 2005 ▶); cell refinement: *SAINT* (Bruker, 2005 ▶); data reduction: *SAINT*; program(s) used to solve structure: *SHELXTL* (Sheldrick, 2008 ▶); program(s) used to refine structure: *SHELXTL*; molecular graphics: *SHELXTL*; software used to prepare material for publication: *SHELXTL* and *PLATON* (Spek, 2009 ▶).

## Supplementary Material

Crystal structure: contains datablocks global, I. DOI: 10.1107/S1600536809014901/ci2786sup1.cif

Structure factors: contains datablocks I. DOI: 10.1107/S1600536809014901/ci2786Isup2.hkl

Additional supplementary materials:  crystallographic information; 3D view; checkCIF report

## Figures and Tables

**Table 1:** Hydrogen-bond geometry (Å, °)

*D*—H⋯*A*	*D*—H	H⋯*A*	*D*⋯*A*	*D*—H⋯*A*
N3*A*—H3*NA*⋯N2*A*^i^	0.90 (2)	2.10 (2)	2.9572 (17)	157 (1)
N3*B*—H3*NB*⋯N2*B*^ii^	0.91 (2)	2.29 (2)	3.0980 (17)	148 (1)
C6*A*—H6*AA*⋯N1*A*^iii^	0.98	2.60	3.4316 (17)	143
C2*B*—H2*BA*⋯*Cg*1	0.93	2.79	3.6350 (14)	151
C20*B*—H20*B*⋯*Cg*2	0.93	2.79	3.4468 (15)	129

Symmetry codes: (i) 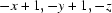; (ii) 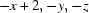; (iii) 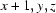. *Cg*1 and *Cg*2 are the centroids of the N2*A*/C1*A*–C5*A* and C7*B*–C12*B* rings, respectively.

**Fig. 1.**
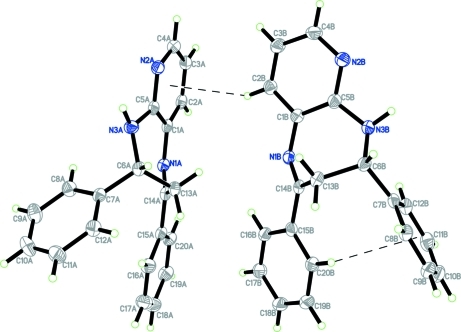
The asymmetric unit of the title compound with atom labels and 50% probability ellipsoids for non-H atoms. C—H···π interactions are shown as dashed lines.

**Fig. 2.**
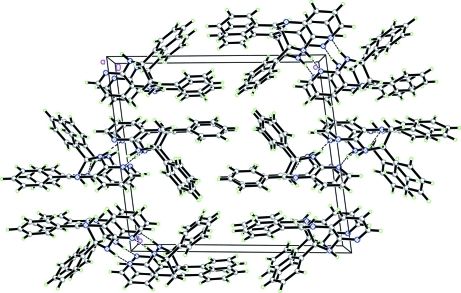
The crystal packing of the title compound, viewed down the a axis, showing centrosymmetric R22(8) dimers. Hydrogen bonds are shown as dashed lines.

## supplementary crystallographic information

### Comment

Pyridodiazepines are bicyclic heterocyclic compounds comprising of pyridine nucleus fused to a seven-membered ring containing two nitrogen atoms (Landquist *et al.*, 1984). These compounds have important role in biological and therapeutic applications (Smalley *et al.*, 1979). 2,4-Diphenyl-4,5-dihydro-3*H*-pyrido[2,3-b][1,4]diazepine was synthesized by our newly developed microwave technique (Goswami *et al.*, 2009). We report here the crystal structure of this compound.

The asymmetric unit of title compound (Fig 1), consists of two crystallographically independent molecules, *A* and *B*. In the molecule *A*, the C7A-C12A and C15A-C20A rings form dihedral angles of 74.12 (7)° and 7.83 (7)°, respectively, with the N2A/C1A-C5A ring, while in *B* these angles are 77.48 (7)° and 21.50 (7)°. The bond lengths (Allen *et al.*, 1987) and angles are within normal ranges. The seven-membered heterocyclic ring adopts a boat conformation.

In the crystal structure, *A*/*A* and *B*/*B* pairs of inversion related molecules are linked by N—H···N hydrogen bonds forming *R*_2_^2^(8) dimers (Fig. 2). The crystal structure is further stabilized by intermolecular C—H···N hydrogen bonds and C—H···π interactions (Table 1).

### Experimental

A mixture of pyridine-2,3-diamine (109 mg, 1.0 mmol) and the chalcone benzylideneacetophenone (208 mg, 1.0 mmol) was thoroughly grinded and taken in an open mouth conical flask and then irradiated at 400 W for 35 min in a microwave oven. The residue was dissolved in water and then extracted with CHCl_3_. The crude product was purified through column chromatography to afford a pure yellow-colored 2,4-diphenyl-4,5-dihydro-3*H*-pyrido[2,3-b][1,4]diazepine. Single crystals were grown by slow evaporation of a chloroform solution (m.p. 126-128 °C).

### Refinement

Atoms H3NA and H3NB were located in a difference Fourier map and refined freely. The remaining H atoms were positioned geometrically and refined using a riding model approximation, with C-H = 0.93-0.98 Å and U_iso_(H) = 1.2U_eq_(C).

### Crystal data

**Table:**

C_20_H_17_N_3_	*Z* = 4
*M**_r_* = 299.37	*F*(000) = 632
Triclinic, *P*1	*D*_x_ = 1.283 Mg m^−^^3^
Hall symbol: -P 1	Mo *K*α radiation, λ = 0.71073 Å
*a* = 5.9969 (3) Å	Cell parameters from 5436 reflections
*b* = 15.3186 (6) Å	θ = 1.3–30.1°
*c* = 17.0676 (7) Å	µ = 0.08 mm^−^^1^
α = 82.588 (3)°	*T* = 100 K
β = 85.266 (2)°	Plate, yellow
γ = 88.670 (2)°	0.50 × 0.33 × 0.05 mm
*V* = 1549.37 (12) Å^3^	

### Data collection

**Table:**

Bruker SMART APEXII CCD area-detector diffractometer	9044 independent reflections
Radiation source: fine-focus sealed tube	5978 reflections with *I* > 2σ(*I*)
graphite	*R*_int_ = 0.053
φ and ω scans	θ_max_ = 30.1°, θ_min_ = 1.3°
Absorption correction: multi-scan (*SADABS*; Bruker, 2005)	*h* = −8→7
*T*_min_ = 0.948, *T*_max_ = 0.996	*k* = −21→21
35167 measured reflections	*l* = −24→23

### Refinement

**Table:**

Refinement on *F*^2^	Primary atom site location: structure-invariant direct methods
Least-squares matrix: full	Secondary atom site location: difference Fourier map
*R*[*F*^2^ > 2σ(*F*^2^)] = 0.054	Hydrogen site location: inferred from neighbouring sites
*wR*(*F*^2^) = 0.133	H atoms treated by a mixture of independent and constrained refinement
*S* = 1.08	*w* = 1/[σ^2^(*F*_o_^2^) + (0.0574*P*)^2^ + 0.0014*P*] where *P* = (*F*_o_^2^ + 2*F*_c_^2^)/3
9044 reflections	(Δ/σ)_max_ = 0.001
423 parameters	Δρ_max_ = 0.30 e Å^−^^3^
0 restraints	Δρ_min_ = −0.25 e Å^−^^3^

### Special details

**Table:**

Experimental. The crystal was placed in the cold stream of an Oxford Cyrosystems Cobra open-flow nitrogen cryostat (Cosier & Glazer, 1986) operating at 100.0 (1)K.
Geometry. All esds (except the esd in the dihedral angle between two l.s. planes) are estimated using the full covariance matrix. The cell esds are taken into account individually in the estimation of esds in distances, angles and torsion angles; correlations between esds in cell parameters are only used when they are defined by crystal symmetry. An approximate (isotropic) treatment of cell esds is used for estimating esds involving l.s. planes.
Refinement. Refinement of F^2^ against ALL reflections. The weighted R-factor wR and goodness of fit S are based on F^2^, conventional R-factors are based on F, with F set to zero for negative F^2^. The threshold expression of F^2^ > 2sigma(F^2^) is used only for calculating R-factors(gt) etc. and is not relevant to the choice of reflections for refinement. R-factors based on F^2^ are statistically about twice as large as those based on F, and R- factors based on ALL data will be even larger.

### Fractional atomic coordinates and isotropic or equivalent isotropic displacement parameters (Å^2^)

**Table:**

	*x*	*y*	*z*	*U*_iso_*/*U*_eq_	
N1A	0.00559 (19)	0.37888 (7)	0.20662 (6)	0.0195 (2)	
N2A	0.2520 (2)	0.42903 (7)	−0.00279 (6)	0.0212 (3)	
N3A	0.3721 (2)	0.48394 (8)	0.10399 (7)	0.0222 (3)	
C1A	0.0532 (2)	0.38179 (8)	0.12384 (8)	0.0186 (3)	
C2A	−0.0998 (2)	0.34139 (8)	0.08381 (8)	0.0205 (3)	
H2AA	−0.2222	0.3134	0.1125	0.025*	
C3A	−0.0752 (2)	0.34166 (9)	0.00250 (8)	0.0218 (3)	
H3AA	−0.1756	0.3130	−0.0238	0.026*	
C4A	0.1050 (2)	0.38634 (9)	−0.03762 (8)	0.0232 (3)	
H4AA	0.1252	0.3867	−0.0923	0.028*	
C5A	0.2287 (2)	0.42889 (8)	0.07678 (8)	0.0191 (3)	
C6A	0.4840 (2)	0.46771 (8)	0.17706 (8)	0.0193 (3)	
H6AA	0.6439	0.4603	0.1619	0.023*	
C7A	0.4611 (2)	0.54539 (8)	0.22395 (8)	0.0192 (3)	
C8A	0.2748 (2)	0.60103 (9)	0.22205 (9)	0.0247 (3)	
H8AA	0.1614	0.5916	0.1902	0.030*	
C9A	0.2555 (3)	0.67030 (10)	0.26686 (9)	0.0313 (4)	
H9AA	0.1302	0.7072	0.2649	0.038*	
C10A	0.4230 (3)	0.68456 (10)	0.31460 (9)	0.0332 (4)	
H10A	0.4096	0.7306	0.3453	0.040*	
C11A	0.6104 (3)	0.63038 (10)	0.31664 (9)	0.0304 (4)	
H11A	0.7236	0.6402	0.3484	0.036*	
C12A	0.6297 (2)	0.56118 (9)	0.27127 (8)	0.0246 (3)	
H12A	0.7565	0.5251	0.2726	0.030*	
C13A	0.4056 (2)	0.38124 (8)	0.22618 (8)	0.0205 (3)	
H13A	0.4367	0.3334	0.1946	0.025*	
H13B	0.4918	0.3701	0.2722	0.025*	
C14A	0.1612 (2)	0.38082 (8)	0.25369 (8)	0.0195 (3)	
C15A	0.0943 (2)	0.37752 (8)	0.33980 (8)	0.0205 (3)	
C16A	0.2301 (3)	0.41094 (9)	0.39090 (8)	0.0267 (3)	
H16A	0.3671	0.4354	0.3711	0.032*	
C17A	0.1636 (3)	0.40818 (10)	0.47101 (9)	0.0320 (4)	
H17A	0.2555	0.4313	0.5043	0.038*	
C18A	−0.0384 (3)	0.37134 (10)	0.50151 (9)	0.0314 (4)	
H18A	−0.0833	0.3698	0.5551	0.038*	
C19A	−0.1734 (3)	0.33676 (10)	0.45153 (9)	0.0290 (3)	
H19A	−0.3087	0.3113	0.4719	0.035*	
C20A	−0.1092 (2)	0.33968 (9)	0.37156 (8)	0.0239 (3)	
H20A	−0.2019	0.3164	0.3386	0.029*	
N3B	0.8455 (2)	0.01768 (8)	0.10185 (7)	0.0262 (3)	
N2B	0.7478 (2)	0.07525 (8)	−0.02255 (7)	0.0282 (3)	
N1B	0.52791 (19)	0.14490 (7)	0.17025 (6)	0.0194 (2)	
C1B	0.5719 (2)	0.14051 (9)	0.08833 (8)	0.0195 (3)	
C2B	0.4391 (2)	0.19151 (9)	0.03704 (8)	0.0225 (3)	
H2BA	0.3329	0.2298	0.0570	0.027*	
C3B	0.4631 (3)	0.18599 (10)	−0.04335 (8)	0.0261 (3)	
H3BA	0.3768	0.2210	−0.0782	0.031*	
C4B	0.6190 (3)	0.12694 (10)	−0.07016 (9)	0.0293 (3)	
H4BA	0.6356	0.1228	−0.1242	0.035*	
C5B	0.7257 (2)	0.08089 (9)	0.05558 (8)	0.0222 (3)	
C6B	0.9974 (2)	0.04096 (8)	0.15846 (8)	0.0205 (3)	
H6BA	1.1475	0.0471	0.1310	0.025*	
C7B	1.0103 (2)	−0.02950 (8)	0.22919 (8)	0.0190 (3)	
C8B	1.1982 (2)	−0.03127 (9)	0.27182 (8)	0.0224 (3)	
H8BA	1.3180	0.0051	0.2528	0.027*	
C9B	1.2092 (3)	−0.08657 (9)	0.34246 (8)	0.0271 (3)	
H9BA	1.3350	−0.0865	0.3709	0.032*	
C10B	1.0341 (3)	−0.14153 (9)	0.37050 (9)	0.0293 (4)	
H10B	1.0398	−0.1777	0.4184	0.035*	
C11B	0.8500 (3)	−0.14265 (9)	0.32713 (9)	0.0290 (3)	
H11B	0.7336	−0.1810	0.3452	0.035*	
C12B	0.8372 (2)	−0.08672 (9)	0.25646 (8)	0.0234 (3)	
H12B	0.7125	−0.0878	0.2276	0.028*	
C13B	0.9294 (2)	0.12981 (8)	0.18612 (8)	0.0205 (3)	
H13C	1.0226	0.1405	0.2276	0.025*	
H13D	0.9585	0.1759	0.1421	0.025*	
C14B	0.6873 (2)	0.13582 (8)	0.21690 (8)	0.0183 (3)	
C15B	0.6238 (2)	0.13550 (8)	0.30306 (8)	0.0196 (3)	
C16B	0.4148 (2)	0.16924 (9)	0.32839 (8)	0.0216 (3)	
H16B	0.3203	0.1946	0.2910	0.026*	
C17B	0.3472 (3)	0.16542 (9)	0.40802 (8)	0.0253 (3)	
H17B	0.2089	0.1889	0.4239	0.030*	
C18B	0.4845 (3)	0.12670 (10)	0.46429 (9)	0.0286 (3)	
H18B	0.4374	0.1229	0.5179	0.034*	
C19B	0.6919 (3)	0.09368 (10)	0.44048 (9)	0.0289 (3)	
H19B	0.7848	0.0678	0.4782	0.035*	
C20B	0.7623 (2)	0.09896 (9)	0.36062 (8)	0.0234 (3)	
H20B	0.9037	0.0778	0.3453	0.028*	
H3NA	0.460 (3)	0.5106 (11)	0.0627 (10)	0.041 (5)*	
H3NB	0.919 (3)	−0.0178 (12)	0.0692 (10)	0.046 (5)*	

### Atomic displacement parameters (Å^2^)

**Table:**

	*U*^11^	*U*^22^	*U*^33^	*U*^12^	*U*^13^	*U*^23^
N1A	0.0186 (6)	0.0177 (5)	0.0215 (6)	0.0010 (5)	−0.0010 (5)	−0.0007 (4)
N2A	0.0226 (6)	0.0190 (5)	0.0216 (6)	0.0006 (5)	−0.0013 (5)	−0.0011 (4)
N3A	0.0261 (7)	0.0216 (6)	0.0188 (6)	−0.0059 (5)	−0.0016 (5)	−0.0008 (5)
C1A	0.0167 (7)	0.0150 (6)	0.0230 (7)	0.0037 (5)	−0.0004 (6)	0.0001 (5)
C2A	0.0164 (7)	0.0167 (6)	0.0271 (7)	0.0025 (5)	0.0001 (6)	0.0003 (5)
C3A	0.0216 (7)	0.0185 (6)	0.0261 (7)	0.0003 (6)	−0.0059 (6)	−0.0032 (5)
C4A	0.0263 (8)	0.0215 (7)	0.0216 (7)	0.0017 (6)	−0.0036 (6)	−0.0016 (5)
C5A	0.0190 (7)	0.0149 (6)	0.0228 (7)	0.0035 (6)	−0.0011 (6)	−0.0014 (5)
C6A	0.0155 (7)	0.0196 (6)	0.0226 (7)	0.0013 (5)	−0.0011 (6)	−0.0026 (5)
C7A	0.0183 (7)	0.0188 (6)	0.0194 (7)	−0.0012 (6)	0.0011 (6)	0.0002 (5)
C8A	0.0206 (7)	0.0251 (7)	0.0290 (8)	0.0025 (6)	−0.0033 (6)	−0.0049 (6)
C9A	0.0251 (8)	0.0272 (8)	0.0422 (9)	0.0028 (7)	0.0018 (7)	−0.0104 (7)
C10A	0.0332 (9)	0.0281 (8)	0.0405 (9)	−0.0039 (7)	0.0044 (8)	−0.0163 (7)
C11A	0.0281 (8)	0.0317 (8)	0.0330 (8)	−0.0067 (7)	−0.0043 (7)	−0.0085 (6)
C12A	0.0204 (7)	0.0237 (7)	0.0299 (8)	−0.0003 (6)	−0.0043 (6)	−0.0026 (6)
C13A	0.0188 (7)	0.0189 (6)	0.0235 (7)	0.0019 (6)	−0.0016 (6)	−0.0023 (5)
C14A	0.0197 (7)	0.0144 (6)	0.0240 (7)	0.0020 (5)	−0.0011 (6)	−0.0013 (5)
C15A	0.0205 (7)	0.0172 (6)	0.0228 (7)	0.0036 (6)	−0.0018 (6)	0.0001 (5)
C16A	0.0269 (8)	0.0267 (7)	0.0262 (8)	−0.0027 (7)	−0.0012 (7)	−0.0019 (6)
C17A	0.0376 (9)	0.0333 (8)	0.0257 (8)	−0.0031 (8)	−0.0036 (7)	−0.0050 (6)
C18A	0.0374 (9)	0.0333 (8)	0.0217 (7)	0.0016 (7)	0.0034 (7)	−0.0011 (6)
C19A	0.0258 (8)	0.0308 (8)	0.0277 (8)	−0.0001 (7)	0.0030 (7)	0.0033 (6)
C20A	0.0211 (7)	0.0242 (7)	0.0254 (7)	0.0026 (6)	−0.0032 (6)	0.0006 (6)
N3B	0.0332 (8)	0.0238 (6)	0.0228 (6)	0.0116 (6)	−0.0083 (6)	−0.0059 (5)
N2B	0.0310 (7)	0.0317 (7)	0.0227 (6)	0.0113 (6)	−0.0060 (6)	−0.0056 (5)
N1B	0.0208 (6)	0.0163 (5)	0.0210 (6)	0.0014 (5)	−0.0024 (5)	−0.0019 (4)
C1B	0.0163 (7)	0.0203 (6)	0.0214 (7)	−0.0002 (6)	−0.0007 (6)	−0.0013 (5)
C2B	0.0192 (7)	0.0228 (7)	0.0256 (7)	0.0032 (6)	−0.0026 (6)	−0.0035 (6)
C3B	0.0241 (8)	0.0290 (7)	0.0249 (7)	0.0059 (6)	−0.0057 (6)	−0.0011 (6)
C4B	0.0319 (9)	0.0366 (8)	0.0199 (7)	0.0097 (7)	−0.0051 (7)	−0.0048 (6)
C5B	0.0218 (7)	0.0213 (6)	0.0235 (7)	0.0022 (6)	−0.0041 (6)	−0.0025 (5)
C6B	0.0189 (7)	0.0209 (6)	0.0210 (7)	0.0035 (6)	−0.0014 (6)	−0.0011 (5)
C7B	0.0191 (7)	0.0166 (6)	0.0214 (7)	0.0031 (6)	0.0004 (6)	−0.0042 (5)
C8B	0.0201 (7)	0.0206 (6)	0.0264 (7)	0.0019 (6)	−0.0018 (6)	−0.0034 (5)
C9B	0.0270 (8)	0.0277 (7)	0.0266 (8)	0.0091 (7)	−0.0063 (7)	−0.0031 (6)
C10B	0.0374 (9)	0.0223 (7)	0.0258 (8)	0.0087 (7)	0.0008 (7)	0.0025 (6)
C11B	0.0305 (9)	0.0193 (7)	0.0354 (8)	−0.0026 (6)	0.0066 (7)	−0.0020 (6)
C12B	0.0211 (7)	0.0197 (6)	0.0297 (8)	0.0017 (6)	−0.0011 (6)	−0.0048 (6)
C13B	0.0183 (7)	0.0181 (6)	0.0242 (7)	0.0001 (6)	−0.0030 (6)	0.0011 (5)
C14B	0.0187 (7)	0.0131 (6)	0.0230 (7)	0.0017 (5)	−0.0024 (6)	−0.0013 (5)
C15B	0.0209 (7)	0.0148 (6)	0.0231 (7)	−0.0012 (6)	−0.0025 (6)	−0.0027 (5)
C16B	0.0215 (7)	0.0196 (6)	0.0235 (7)	0.0010 (6)	−0.0041 (6)	−0.0013 (5)
C17B	0.0228 (8)	0.0265 (7)	0.0267 (7)	0.0030 (6)	−0.0014 (6)	−0.0044 (6)
C18B	0.0333 (9)	0.0318 (8)	0.0200 (7)	0.0057 (7)	−0.0004 (7)	−0.0031 (6)
C19B	0.0320 (9)	0.0311 (8)	0.0242 (7)	0.0092 (7)	−0.0078 (7)	−0.0039 (6)
C20B	0.0212 (7)	0.0238 (7)	0.0255 (7)	0.0048 (6)	−0.0035 (6)	−0.0042 (6)

### Geometric parameters (Å, °)

**Table:**

N1A—C14A	1.2838 (17)	N3B—C5B	1.3933 (17)
N1A—C1A	1.4134 (16)	N3B—C6B	1.4632 (18)
N2A—C4A	1.3309 (17)	N3B—H3NB	0.910 (18)
N2A—C5A	1.3535 (16)	N2B—C4B	1.3391 (18)
N3A—C5A	1.3665 (17)	N2B—C5B	1.3428 (17)
N3A—C6A	1.4553 (17)	N1B—C14B	1.2875 (16)
N3A—H3NA	0.905 (17)	N1B—C1B	1.4113 (16)
C1A—C2A	1.3895 (19)	C1B—C2B	1.3857 (19)
C1A—C5A	1.4207 (19)	C1B—C5B	1.4157 (19)
C2A—C3A	1.3828 (19)	C2B—C3B	1.3810 (19)
C2A—H2AA	0.93	C2B—H2BA	0.93
C3A—C4A	1.377 (2)	C3B—C4B	1.378 (2)
C3A—H3AA	0.93	C3B—H3BA	0.93
C4A—H4AA	0.93	C4B—H4BA	0.93
C6A—C7A	1.5151 (18)	C6B—C7B	1.5177 (18)
C6A—C13A	1.5349 (18)	C6B—C13B	1.5330 (18)
C6A—H6AA	0.98	C6B—H6BA	0.98
C7A—C12A	1.3888 (19)	C7B—C12B	1.3858 (19)
C7A—C8A	1.3894 (19)	C7B—C8B	1.3888 (19)
C8A—C9A	1.3833 (19)	C8B—C9B	1.3865 (19)
C8A—H8AA	0.93	C8B—H8BA	0.93
C9A—C10A	1.383 (2)	C9B—C10B	1.377 (2)
C9A—H9AA	0.93	C9B—H9BA	0.93
C10A—C11A	1.381 (2)	C10B—C11B	1.381 (2)
C10A—H10A	0.93	C10B—H10B	0.93
C11A—C12A	1.388 (2)	C11B—C12B	1.393 (2)
C11A—H11A	0.93	C11B—H11B	0.93
C12A—H12A	0.93	C12B—H12B	0.93
C13A—C14A	1.5017 (19)	C13B—C14B	1.5075 (19)
C13A—H13A	0.97	C13B—H13C	0.97
C13A—H13B	0.97	C13B—H13D	0.97
C14A—C15A	1.4858 (18)	C14B—C15B	1.4878 (18)
C15A—C16A	1.3921 (19)	C15B—C20B	1.3924 (19)
C15A—C20A	1.401 (2)	C15B—C16B	1.4012 (19)
C16A—C17A	1.388 (2)	C16B—C17B	1.3801 (19)
C16A—H16A	0.93	C16B—H16B	0.93
C17A—C18A	1.382 (2)	C17B—C18B	1.383 (2)
C17A—H17A	0.93	C17B—H17B	0.93
C18A—C19A	1.384 (2)	C18B—C19B	1.382 (2)
C18A—H18A	0.93	C18B—H18B	0.93
C19A—C20A	1.3826 (19)	C19B—C20B	1.3858 (19)
C19A—H19A	0.93	C19B—H19B	0.93
C20A—H20A	0.93	C20B—H20B	0.93
			
C14A—N1A—C1A	121.82 (12)	C5B—N3B—C6B	122.33 (11)
C4A—N2A—C5A	119.50 (12)	C5B—N3B—H3NB	108.2 (11)
C5A—N3A—C6A	126.75 (11)	C6B—N3B—H3NB	109.2 (11)
C5A—N3A—H3NA	109.3 (10)	C4B—N2B—C5B	118.79 (13)
C6A—N3A—H3NA	113.2 (11)	C14B—N1B—C1B	120.74 (12)
C2A—C1A—N1A	116.30 (12)	C2B—C1B—N1B	117.63 (12)
C2A—C1A—C5A	116.85 (12)	C2B—C1B—C5B	117.56 (12)
N1A—C1A—C5A	126.54 (12)	N1B—C1B—C5B	124.45 (12)
C3A—C2A—C1A	121.89 (13)	C3B—C2B—C1B	120.67 (13)
C3A—C2A—H2AA	119.1	C3B—C2B—H2BA	119.7
C1A—C2A—H2AA	119.1	C1B—C2B—H2BA	119.7
C4A—C3A—C2A	116.85 (13)	C4B—C3B—C2B	117.80 (13)
C4A—C3A—H3AA	121.6	C4B—C3B—H3BA	121.1
C2A—C3A—H3AA	121.6	C2B—C3B—H3BA	121.1
N2A—C4A—C3A	123.86 (13)	N2B—C4B—C3B	123.50 (13)
N2A—C4A—H4AA	118.1	N2B—C4B—H4BA	118.2
C3A—C4A—H4AA	118.1	C3B—C4B—H4BA	118.2
N2A—C5A—N3A	113.73 (12)	N2B—C5B—N3B	115.02 (12)
N2A—C5A—C1A	120.92 (12)	N2B—C5B—C1B	121.65 (12)
N3A—C5A—C1A	125.04 (12)	N3B—C5B—C1B	122.96 (12)
N3A—C6A—C7A	111.98 (11)	N3B—C6B—C7B	112.44 (11)
N3A—C6A—C13A	110.97 (11)	N3B—C6B—C13B	110.60 (11)
C7A—C6A—C13A	112.71 (11)	C7B—C6B—C13B	110.36 (11)
N3A—C6A—H6AA	106.9	N3B—C6B—H6BA	107.8
C7A—C6A—H6AA	106.9	C7B—C6B—H6BA	107.8
C13A—C6A—H6AA	106.9	C13B—C6B—H6BA	107.8
C12A—C7A—C8A	118.64 (12)	C12B—C7B—C8B	118.99 (12)
C12A—C7A—C6A	119.28 (12)	C12B—C7B—C6B	123.16 (13)
C8A—C7A—C6A	122.08 (12)	C8B—C7B—C6B	117.63 (12)
C9A—C8A—C7A	120.96 (14)	C9B—C8B—C7B	120.76 (13)
C9A—C8A—H8AA	119.5	C9B—C8B—H8BA	119.6
C7A—C8A—H8AA	119.5	C7B—C8B—H8BA	119.6
C10A—C9A—C8A	119.83 (15)	C10B—C9B—C8B	120.02 (14)
C10A—C9A—H9AA	120.1	C10B—C9B—H9BA	120.0
C8A—C9A—H9AA	120.1	C8B—C9B—H9BA	120.0
C11A—C10A—C9A	119.97 (14)	C9B—C10B—C11B	119.70 (13)
C11A—C10A—H10A	120.0	C9B—C10B—H10B	120.2
C9A—C10A—H10A	120.0	C11B—C10B—H10B	120.2
C10A—C11A—C12A	120.03 (15)	C10B—C11B—C12B	120.51 (14)
C10A—C11A—H11A	120.0	C10B—C11B—H11B	119.7
C12A—C11A—H11A	120.0	C12B—C11B—H11B	119.7
C11A—C12A—C7A	120.56 (14)	C7B—C12B—C11B	119.94 (14)
C11A—C12A—H12A	119.7	C7B—C12B—H12B	120.0
C7A—C12A—H12A	119.7	C11B—C12B—H12B	120.0
C14A—C13A—C6A	113.96 (11)	C14B—C13B—C6B	114.07 (11)
C14A—C13A—H13A	108.8	C14B—C13B—H13C	108.7
C6A—C13A—H13A	108.8	C6B—C13B—H13C	108.7
C14A—C13A—H13B	108.8	C14B—C13B—H13D	108.7
C6A—C13A—H13B	108.8	C6B—C13B—H13D	108.7
H13A—C13A—H13B	107.7	H13C—C13B—H13D	107.6
N1A—C14A—C15A	117.83 (12)	N1B—C14B—C15B	117.11 (12)
N1A—C14A—C13A	123.05 (12)	N1B—C14B—C13B	122.04 (12)
C15A—C14A—C13A	119.03 (12)	C15B—C14B—C13B	120.80 (12)
C16A—C15A—C20A	118.30 (13)	C20B—C15B—C16B	118.03 (12)
C16A—C15A—C14A	121.62 (13)	C20B—C15B—C14B	122.05 (13)
C20A—C15A—C14A	120.07 (12)	C16B—C15B—C14B	119.86 (12)
C17A—C16A—C15A	120.80 (14)	C17B—C16B—C15B	120.97 (13)
C17A—C16A—H16A	119.6	C17B—C16B—H16B	119.5
C15A—C16A—H16A	119.6	C15B—C16B—H16B	119.5
C18A—C17A—C16A	120.34 (15)	C16B—C17B—C18B	120.17 (14)
C18A—C17A—H17A	119.8	C16B—C17B—H17B	119.9
C16A—C17A—H17A	119.8	C18B—C17B—H17B	119.9
C17A—C18A—C19A	119.41 (14)	C19B—C18B—C17B	119.69 (14)
C17A—C18A—H18A	120.3	C19B—C18B—H18B	120.2
C19A—C18A—H18A	120.3	C17B—C18B—H18B	120.2
C20A—C19A—C18A	120.67 (14)	C18B—C19B—C20B	120.29 (14)
C20A—C19A—H19A	119.7	C18B—C19B—H19B	119.9
C18A—C19A—H19A	119.7	C20B—C19B—H19B	119.9
C19A—C20A—C15A	120.48 (14)	C19B—C20B—C15B	120.81 (14)
C19A—C20A—H20A	119.8	C19B—C20B—H20B	119.6
C15A—C20A—H20A	119.8	C15B—C20B—H20B	119.6
			
C14A—N1A—C1A—C2A	152.11 (12)	C14B—N1B—C1B—C2B	−147.76 (13)
C14A—N1A—C1A—C5A	−34.49 (19)	C14B—N1B—C1B—C5B	39.29 (19)
N1A—C1A—C2A—C3A	178.31 (12)	N1B—C1B—C2B—C3B	−175.65 (12)
C5A—C1A—C2A—C3A	4.25 (19)	C5B—C1B—C2B—C3B	−2.2 (2)
C1A—C2A—C3A—C4A	−1.9 (2)	C1B—C2B—C3B—C4B	1.4 (2)
C5A—N2A—C4A—C3A	0.8 (2)	C5B—N2B—C4B—C3B	−0.2 (2)
C2A—C3A—C4A—N2A	−0.7 (2)	C2B—C3B—C4B—N2B	−0.2 (2)
C4A—N2A—C5A—N3A	−172.10 (12)	C4B—N2B—C5B—N3B	172.52 (13)
C4A—N2A—C5A—C1A	1.77 (19)	C4B—N2B—C5B—C1B	−0.7 (2)
C6A—N3A—C5A—N2A	−142.34 (13)	C6B—N3B—C5B—N2B	127.54 (14)
C6A—N3A—C5A—C1A	44.1 (2)	C6B—N3B—C5B—C1B	−59.4 (2)
C2A—C1A—C5A—N2A	−4.16 (19)	C2B—C1B—C5B—N2B	1.8 (2)
N1A—C1A—C5A—N2A	−177.54 (12)	N1B—C1B—C5B—N2B	174.79 (13)
C2A—C1A—C5A—N3A	168.99 (12)	C2B—C1B—C5B—N3B	−170.78 (13)
N1A—C1A—C5A—N3A	−4.4 (2)	N1B—C1B—C5B—N3B	2.2 (2)
C5A—N3A—C6A—C7A	−131.74 (14)	C5B—N3B—C6B—C7B	149.17 (13)
C5A—N3A—C6A—C13A	−4.83 (19)	C5B—N3B—C6B—C13B	25.30 (18)
N3A—C6A—C7A—C12A	−149.95 (13)	N3B—C6B—C7B—C12B	−27.20 (18)
C13A—C6A—C7A—C12A	84.08 (15)	C13B—C6B—C7B—C12B	96.80 (15)
N3A—C6A—C7A—C8A	30.91 (18)	N3B—C6B—C7B—C8B	158.13 (12)
C13A—C6A—C7A—C8A	−95.05 (15)	C13B—C6B—C7B—C8B	−77.86 (15)
C12A—C7A—C8A—C9A	−0.7 (2)	C12B—C7B—C8B—C9B	−2.8 (2)
C6A—C7A—C8A—C9A	178.48 (13)	C6B—C7B—C8B—C9B	172.11 (12)
C7A—C8A—C9A—C10A	−0.2 (2)	C7B—C8B—C9B—C10B	1.0 (2)
C8A—C9A—C10A—C11A	0.8 (2)	C8B—C9B—C10B—C11B	1.4 (2)
C9A—C10A—C11A—C12A	−0.5 (2)	C9B—C10B—C11B—C12B	−1.9 (2)
C10A—C11A—C12A—C7A	−0.4 (2)	C8B—C7B—C12B—C11B	2.2 (2)
C8A—C7A—C12A—C11A	1.0 (2)	C6B—C7B—C12B—C11B	−172.36 (13)
C6A—C7A—C12A—C11A	−178.18 (13)	C10B—C11B—C12B—C7B	0.1 (2)
N3A—C6A—C13A—C14A	−63.00 (15)	N3B—C6B—C13B—C14B	52.40 (15)
C7A—C6A—C13A—C14A	63.51 (15)	C7B—C6B—C13B—C14B	−72.66 (14)
C1A—N1A—C14A—C15A	179.96 (11)	C1B—N1B—C14B—C15B	−176.09 (11)
C1A—N1A—C14A—C13A	−3.49 (19)	C1B—N1B—C14B—C13B	6.60 (18)
C6A—C13A—C14A—N1A	70.57 (16)	C6B—C13B—C14B—N1B	−74.94 (15)
C6A—C13A—C14A—C15A	−112.93 (13)	C6B—C13B—C14B—C15B	107.84 (13)
N1A—C14A—C15A—C16A	−155.81 (13)	N1B—C14B—C15B—C20B	158.89 (12)
C13A—C14A—C15A—C16A	27.50 (18)	C13B—C14B—C15B—C20B	−23.76 (18)
N1A—C14A—C15A—C20A	24.54 (18)	N1B—C14B—C15B—C16B	−18.26 (18)
C13A—C14A—C15A—C20A	−152.15 (13)	C13B—C14B—C15B—C16B	159.09 (12)
C20A—C15A—C16A—C17A	−1.1 (2)	C20B—C15B—C16B—C17B	−0.8 (2)
C14A—C15A—C16A—C17A	179.25 (13)	C14B—C15B—C16B—C17B	176.51 (12)
C15A—C16A—C17A—C18A	0.6 (2)	C15B—C16B—C17B—C18B	−1.0 (2)
C16A—C17A—C18A—C19A	0.4 (2)	C16B—C17B—C18B—C19B	1.5 (2)
C17A—C18A—C19A—C20A	−0.7 (2)	C17B—C18B—C19B—C20B	−0.2 (2)
C18A—C19A—C20A—C15A	0.2 (2)	C18B—C19B—C20B—C15B	−1.6 (2)
C16A—C15A—C20A—C19A	0.7 (2)	C16B—C15B—C20B—C19B	2.0 (2)
C14A—C15A—C20A—C19A	−179.64 (13)	C14B—C15B—C20B—C19B	−175.17 (12)

### Hydrogen-bond geometry (Å, °)

**Table:**

*D*—H···*A*	*D*—H	H···*A*	*D*···*A*	*D*—H···*A*
N3A—H3NA···N2A^i^	0.90 (2)	2.10 (2)	2.9572 (17)	157 (1)
N3B—H3NB···N2B^ii^	0.91 (2)	2.29 (2)	3.0980 (17)	148 (1)
C6A—H6AA···N1A^iii^	0.98	2.60	3.4316 (17)	143
C2B—H2BA···Cg1	0.93	2.79	3.6350 (14)	151
C20B—H20B···Cg2	0.93	2.79	3.4468 (15)	129

Symmetry codes: (i) −*x*+1, −*y*+1, −*z*; (ii) −*x*+2, −*y*, −*z*; (iii) *x*+1, *y*, *z*.
